# Accuracy Validation of the Elecsys HBsAg II Quant Assay and Its Utility in Resolving Equivocal Qualitative HBsAg Results

**DOI:** 10.3390/medicina59030443

**Published:** 2023-02-23

**Authors:** Jaehyeon Lee, Seung Yeob Lee, Yong Gon Cho, Dal Sik Kim, Joonhong Park

**Affiliations:** 1 Department of Laboratory Medicine, Jeonbuk National University Medical School and Hospital, Jeonju 54907, Republic of Korea; 2 Research Institute of Clinical Medicine, Jeonbuk National University, Jeonju 54907, Republic of Korea; 3 Biomedical Research Institute, Jeonbuk National University Hospital, Jeonju 54907, Republic of Korea

**Keywords:** accuracy validation, hepatitis B surface antigen, Elecsys HBsAg II Quant assay, ADVIA Centaur HBsAg II, HBsAg quantification, equivocal HBsAg result

## Abstract

*Background and Objectives*: There are reports of false qualitative HBsAg results, because of various causes, such as samples with low HBsAg concentrations that may produce false positives. The main aims of this study were to validate the analytical accuracy and to assess the utility of the Elecsys assay compared to that of the qualitative HbsAg assay as a screening test in resolving equivocal qualitative HbsAg results. *Materials and Methods:* The limit of blank (LoB), the limit of detection (LoD), the limit of quantification (LoQ), and linearity were estimated to validate the analytical accuracy of the Elecsys HBsAg II Quant assay. A total of 449 serum samples showing initial equivocal results (1–50 index) were evaluated by Elecsys HBsAg II Quant and ADVIA Centaur HBsAg II assays. *Results:* The LoQ of the assay was determined to be 0.050 IU/mL, as provided by the manufacturer. The Kappa agreement between the two assays was almost perfect, at 0.9669, despite seven discordant results. With a specificity of 100% at new cut-off index value ≥5.42, about 78 samples (17%, 78/449) with index value ≥5.42 were interpreted as positives without further duplicate tests, however the remaining 371 samples with index value <5.42 need to be confirmed with additional HBV marker assays. *Conclusions:* We confirm that the Elecsys HBsAg II Quant assay is accurate and sensitive for HBV infection and recommend it as an alternative confirmatory HBsAg assay for resolving equivocal qualitative HBsAg results.

## 1. Introduction

The hepatitis B surface antigen (HBsAg), a component of the external envelope of the hepatitis B virus (HBV), is often considered the first immunological marker of HBV infection [1]. HBsAg quantification is valuable for identifying patients who are able to achieve sustained conditions after the termination of therapy [2] and response monitoring for nucleoside/nucleotide analog (NUC) treatment [3]. It can also be used for monitoring HBsAg levels along with the HBV DNA, which may help implement response-guided peginterferon therapy and achieve optimal prognosis based on sustained HBsAg loss with or without seroconversion to anti-HBs [4]. During antiviral therapy with NUCs, HBsAg levels decrease, reflecting improved control of the virus by the host’s immune system, whereas lower HBsAg levels at the end of the antiviral therapy are related to continued remission [5]. To date, automated and highly sensitive immunoassays have been developed for HBsAg quantification, and several commercial immunoassays have been compared and widely used in medical laboratories [6,7,8]. Among these, the Elecsys HBsAg II Quant assay (Roche Diagnostics, Mannheim, Germany) is individually sufficient for quantifying serum HBsAg levels, with significantly high correlation and precision compared to other immunoassays regardless of the serum HBsAg levels, chronic hepatitis B (CHB) infection status, HBV-carrying drug-resistant mutations, HBV genotypes, and immunosuppression levels [6,9,10]. In accordance with the application criteria for public health insurance in the Korea, immunoassays for the in vitro quantitative determination of HBsAg are available only to individuals with a confirmed HBsAg-positive status. Therefore, qualitative HBsAg immunoassays are mostly limited to the diagnosis of HBV in clinical practice [1]. However, there are reports of false qualitative HBsAg results because of various causes [11,12,13,14], such as samples with low HBsAg concentrations that may produce false positives.

The main aims of this study were to validate the analytical accuracy and operational cutoff value of the Elecsys HBsAg II Quant assay that could be used to identify the presence of HBsAg as a quantitative marker of HBV infection. The secondary goal was to assess the utility of the Elecsys assay compared to that of the qualitative HbsAg assay as a screening test in resolving equivocal qualitative HbsAg results in the medical laboratory.

## 2. Materials and Methods

### 2.1. Validation of the Analytical Accuracy

To validate the analytical accuracy of the Elecsys HbsAg II Quant assay, we first estimated the limit of blank (LoB), the limit of detection (LoD), the limit of quantification (LoQ), and linearity. To validate the LoB of the assay, we measured a total of 60 replicates of the blanks using 20 replicates per five blanks, once a day, for three consecutive days. To validate the LoD and the LoQ according to the CLSI EP17-A2 (Evaluation of Detection Capability for Clinical Laboratory Measurement Procedures) guideline, we tested six serial dilutions from the World Health Organization (WHO) 3rd international standard (IS) reference material and HBSAGQ2 Dil HepB as the diluent (Roche Diagnostics, Indianapolis, IN, USA) for HBsAg in 20 replicates per five samples, once daily, for three consecutive days. The six serial dilutions were 0.250, 0.200, 0.150, 0.100, 0.070, and 0.050 IU/mL. The lowest concentration at which the coefficient of variation (CV, %) was less than or equal to the total allowable error (TE) was considered the LoQ. To validate the linearity of the assay according to the CLSI EP06-A (Evaluation of Linearity of Quantitative Measurement Procedures) guideline, five expected diluted assayed samples—that is, 0.01 (dilution ratio of minimum to maximum value = 4:0), 11.90 (3:1), 23.70 (2:2), 35.50 (1:3), and 47.30 IU/mL (0:4)—were prepared within the concentration of the WHO 3rd IS (47.3 IU/mL) and measured for five replicates at each level. The LoQ accuracy goal was a TE <20%. All assays were performed with the Elecsys e601 (Roche Diagnostics) as per the manufacturer’s recommendation, and a quantitative HBsAg result <0.050 IU/mL was considered to be negative when compared with the qualitative HBsAg result.

### 2.2. Comparative Evaluation of Elecsys HBsAg II Quant and ADVIA Centaur HBsAg II Assays

We collected a total of 449 residual serum samples left over from routine qualitative HBsAg and anti-HBs antibody (Ab) tests at the department of laboratory medicine of Jeonbuk National University Hospital (Jeonju, Republic of Korea) between November 2018 and January 2022. These samples, ranging from 1 to 50 index for the initial HBsAg test, were selected. According to the manufacturer’s recommendation, the sample is positive for HBsAg when the sample is greater than 50 or flagged as >index range, and no further testing is required. However, if the samples show initial equivocal results ranging from 1 to 50 index, further confirmatory testing is required, even though they were presumed to be positive with an index ≥1.0 from the measurements obtained using the ADVIA Centaur HBsAg II assay with ADVIA Centaur XP (Siemens Diagnostics, Tarrytown, NY, USA). To determine whether the status was positive or negative, we performed additional tests as duplicates. Briefly, if 2 out of 3 results had index value <1.0, the sample was considered negative for HBsAg. If at least 2 of the 3 results had index value ≥1.0, the sample was repeatedly positive, and the presence of HBsAg was confirmed via the ADVIA Centaur HBsAg Confirmatory assay (Siemens Diagnostics). Equivocal samples remaining after the qualitative HBsAg testing were measured immediately using the Elecsys HBsAg II Quant assay, a two-step sandwich electrochemiluminescence immunoassay (ECLIA) with improved sensitivity for the in vitro quantitative determination of HBsAg in confirmed HBsAg-positive samples. We generated the results from the Elecsys HBsAg II Quant assay in terms of the cutoff index (COI) and converted them into the WHO IU/L standard using the following conversion factor for estimating HBsAg units, as advised by the manufacturer: 1 WHO IU/L = 18.21 COI. Table 1 presents the performance characteristics of the two HBsAg assays provided by the manufacturer. For all study patients, we regularly reviewed the clinical history and additional laboratory results, including anti-HBs Ab, HBeAg, anti-HBe Ab, and anti-HBc IgG/IgM Ab tests as well as HBV DNA quantitation associated with HBV, using the electronic medical record (EMR) and the laboratory information system (LIS) at 1, 3, 6, and 12 months after the initial HBsAg test. Particularly, the seven individuals with the discordant HBsAg results were followed up with, and additional serologic anti-HBs Ab, HBeAg, anti-HBe Ab, and anti-HBc IgG/IgM Ab tests, as well as HBV DNA quantitation, were performed at 1, 3, 6, and 12 months after the initial HBsAg test.

### 2.3. Statistical Analysis

We calculated the qualitative interrater agreement between the two assays and interpreted it according to the guidelines of Bland and Altman. In particular, values in the range of 0.81–1 indicated almost perfect agreement. We assessed the diagnostic performance of the ADVIA Centaur HBsAg II in determining a new COI value as the “putative positive HBsAg” using the area under the receiver operating characteristic (ROC) curve (AUC). We performed statistical analyses using MedCalc version 17.6 (MedCalc, Ostend, Belgium), where we considered *p* values <0.05 to be statistically significant. The sensitivity, specificity, positive and negative likelihood ratios, positive predictive value (PPV) and negative predictive value (NPV), and accuracy were calculated using the diagnostic test evaluation calculator provided by MedCalc (https://www.medcalc.org/calc/diagnostic_test.php, accessed on 3 December 2022). Concordant results between two assays for 167 positives and 275 negatives were considered as true positive and true negative, respectively. Three false negatives and four false positives were classified based on patients’ electronic medical records and additional serologic anti-HBs, HBeAg, anti-HBe, and anti-HBc IgG/IgM tests (Roche Diagnostics) as well as HBV DNA quantitation were carried out. Sensitivity was defined as true positive/(true positive + false negative). Specificity was defined as true negative/(false positive + true negative). PPV was defined as (sensitivity × prevalence)/(sensitivity × prevalence + (1 − specificity) × (1 − prevalence)). NPV was defined as (specificity × (1 − prevalence))/((1 − sensitivity) × prevalence + specificity × (1 − prevalence)). Accuracy was defined as sensitivity × prevalence + specificity × (1 − prevalence).

## 3. Results

### 3.1. Analytical Accuracy of the Elecsys HBsAg II Quant Assay

**Figure 1:**
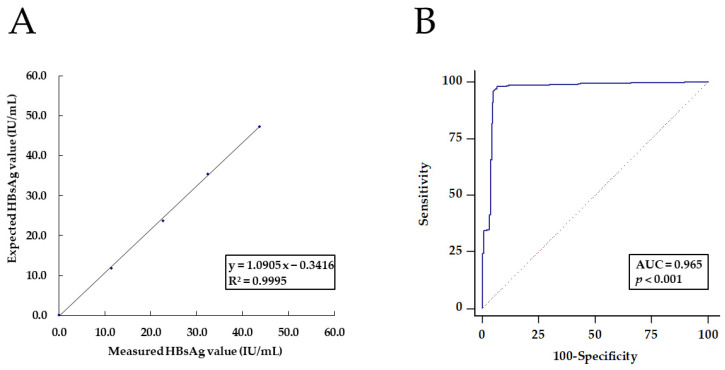
(**A**) Linearity analysis of the Elecsys HBsAg II Quant assay using five expected diluted assayed samples. The best-fit regression equation for the Elecsys HBsAg II Quant assay was y = 1.0905x − 0.3416. Abbreviation: HBsAg, hepatitis B surface antigen. (**B**) The receiver operating characteristic (ROC) curve analysis of the ADVIA Centaur HBsAg II assay, confirmed with the ADVIA Centaur HBsAg Confirmatory assay without further duplicate tests, shows the area under the curve to be 0.965.

After sorting the blank results from the lowest to the highest, we calculated the 95th percentile for the distribution of the blank sample results (defined as the LoB of the Elecsys HBsAg II Quant assay) corresponding to the desired risk probability of the type I error (α = 0.05). After a parametric analysis, the LoD was calculated because the variability in the measured results was relatively consistent across the low-level samples. Unfortunately, because the raw results were not quantitatively measured and only those that yielded <0.050 IU/mL were considered, the LoB and the LoD of the Elecsys HBsAg II Quant assay could not be determined. As per the manufacturer’s instruction, the LoB and the LoD were 0.03 IU/mL and 0.05 IU/mL, respectively. The CV range of the LoQ of the Elecsys HBsAg II Quant assay varied from 5.9% at 0.05 IU/mL to 17.3% at 0.100 IU/mL, where the values satisfied the condition of being less than the accuracy goal of 20% for the TE. Thus, the LoQ of the assay was determined to be 0.050 IU/mL, as provided by the manufacturer. In a linearity analysis of the dataset comprising the five assayed levels, the HBsAg values measured by the Elecsys HBsAg II Quant assay were correlated with the expected assayed HBsAg levels ranging from 0.100 to 47.30 IU/mL for quantification. The Elecsys HBsAg II Quant assay yielded a mean of 43.64 IU/mL and a 92.3% recovery rate when the WHO 3rd IS was 47.30 IU/mL. The linear coefficient was found through regression analysis (R^2^ = 0.9995; *p* < 0.001); the best-fit regression equation for the Elecsys HBsAg II Quant assay was y = 1.0905x − 0.3416 (Figure 1A).

### 3.2. Comparative Evaluation for Resolving the Equivocal Qualitative HBsAg Results

In the comparative qualitative evaluation between the two assays for resolving the equivocal qualitative HBsAg results, we tested a total of 449 equivocal samples near the cutoff level. Among the 449 samples, there were 442 concordant and 7 discordant results between the two assays. Among the 442 concordant results, there were 275 negatives (2 out of 3 results had index value <1.0) as per the ADVIA Centaur HBsAg II and <0.05 as per the Elecsys HBsAg II Quant assays. The remaining 167 results were positive (2 out of 3 results had index value ≥1.0) as per the ADVIA Centaur HBsAg II and ≥0.05 as per the Elecsys HBsAg II Quant assays. According to the review of the EMR and laboratory findings during the follow-up period, among the 275 HbsAg-negative samples, 243 samples were positive for anti-HBs Ab and/or anti-HBc IgG Ab. The remaining 32 samples were negative for anti-HBs Ab and/or anti-HBc IgG Ab. All HBsAg-negative patients visited to receive outpatient treatment, a general health checkup, or elective general surgery but did not receive medical service or treatment for HBV infection. Among the 167 HBsAg-positive samples, 153 samples were negative for anti-HBs Ab, had been diagnosed as CHB, and the patients visited regularly to receive outpatient antiviral therapy. The HBV DNA level was regularly monitored using an HBV DNA quantitation test every three months. The remaining 14 patients were referred to the outpatient department of gastroenterology of Jeonbuk National University Hospital and diagnosed as HBV carriers. An HBV DNA quantitation test confirmed the presence of HBV.

To determine the assay that produced the false results for the seven discordant results, we reviewed the patients’ electronic medical records and carried out additional serologic anti-HBs, HBeAg, anti-HBe, and anti-HBc IgG/IgM tests (Roche Diagnostics) as well as HBV DNA quantitation (COBAS AmpliPrep/COBAS TaqMan HBV Test, v2.0, Roche Diagnostics). From these, three false negatives and four false positives were obtained through qualitative measurements provided by the ADVIA Centaur HBsAg II assay. Three outpatients (sample IDs 271, 285, and 299 in Table 2) who were previously diagnosed with CHB showed qualitative false-negative HBsAg results of 0.05, 116.04, and 0.80 IU/mL, as quantitatively measured by the Elecsys HBsAg II Quant assay. Four outpatients (sample IDs 28, 257, 309, and 362 in Table 2) who were diagnosed to have acute hepatitis C (AHC), acute cholangitis with common bile duct stones, bronchiectasis and emphysema, and latent pulmonary tuberculosis, respectively, showed qualitative false-positive HBsAg levels <0.05 IU/mL, as quantitatively measured by the Elecsys HBsAg II Quant assay. An HBV DNA quantitation test was performed in six out of the seven discordant HBsAg samples, and all six were negative for HBV DNA (described as “ND”). One sample (ID 285) was not available for HBV DNA quantitation. However, according to the EMR review and the serologic test, it was diagnosed as CHB. Table 2 presents the detailed serologic test results of the seven samples showing discordant HBsAg values measured by the two assays.

The Kappa agreement between the two assays was almost perfect, at 0.9669, despite the seven discordant results (three by ADVIA Centaur HBsAg II Negative and Elecsys HBsAg II Quant Positive; four by ADVIA Centaur HBsAg II Positive and Elecsys HBsAg II Quant Negative). The concordance rate and accuracy of the ADVIA Centaur HBsAg II assay relative to the Elecsys HBsAg II Quant assay from the confirmed results were 98.44% and 98.55%, respectively, when the HBsAg seroprevalence of 5.3% from the Western Pacific region, including the Korea, was applied [15]. Table 3 summarizes the diagnostic performance statistics of the two assays.

Further, to reduce the labor burden from duplicate qualitative HBsAg testing for equivocal results from the initial HBsAg tests, we assessed a new COI value for the ADVIA Centaur HBsAg II assay. The highest index of the negatively confirmed sample by the ADVIA Centaur HBsAg II assay was 3.63. The ROC curve analysis of this assay showed an AUC of 0.965 (*p* < 0.001), with a specificity of 100% (95% CI, 98.4–100) and a sensitivity of 24.15% (95% CI, 19.8–29.0) at the new COI value of 5.42 (Figure 1B). Accordingly, only those HBsAg samples with index value <5.42 needed to be confirmed with an approved method, such as the ADVIA Centaur HBsAg Confirmatory assay or via additional HBV marker assays without further duplicate tests.

## 4. Discussion

Detecting quantitative HBsAg is fairly easy and inexpensive and quantitative HBsAg correlated well with the serum HBV DNA level [1]. Serum HBV DNA quantitation is relatively expensive but not yet readily available in some areas, although it is the gold standard for monitoring viral load [16]. The Elecsys HBsAg II Quant assay is fully able to quantify the serum HBsAg level in CHB, with high correlation and accuracy compared to other automated immunoassays [9,10,17,18]. It also shows good performance in terms of precision, linearity, carryover rate, and specificity. Moreover, the HBsAg levels at baseline, 12 weeks, and 24 weeks after the start of treatment are useful for predicting the virologic response in patients with CHB infection [18]. Serum HBsAg levels not only show strong correlations with other virological markers, including serum intrahepatic covalently closed circular DNA (cccDNA) and HBV DNA in the HBeAg-positive phase, but also weak correlations in the HBeAg-negative phase [19].

Knowledge of the approximate prevalence of a certain disease is a prerequisite for interpreting screening test results [20]. As the prevalence of a certain disease decreases, the positive predictive value (PPV) of the screening test also decreases; however, false positivity increases if all other parameters are constant [21]. The Korea is an intermediate endemic country for HBV infections, with an estimated prevalence of approximately 3% according to the 2016 Korea National Health and Nutrition Examination Survey (KNHANES) [22]. However, remarkable progress has been achieved in the management of CHB infection after the implementation of HBV vaccination, along with nationwide screening and advances in antiviral therapy [23]. The HBsAg positivity rates in younger age groups (<18 years) have markedly declined over the years, from 2.2% in 1998, 1.9% in 2001, and 1.9% in 2007 to 0.3% in 2016. The prevalence of CHB in Korean children has met the WHO interim 2020 target of 1% in children aged 5 years and is projected to decline to 0.1% by 2030 [22]. Thus, screening tests for HBV infection favor specificity over sensitivity, since further assessments of the persons testing positive are costly and labor intensive.

In low-prevalence situations, the single-assay testing strategy may result in considerably low PPV and false-positive results. The reduced diagnostic accuracy of a single test may result in false-positive (or, less often, false-negative) reporting. False positives can increase costs and lead to unnecessary procedures associated with follow-up tests and clinical evaluations, whereas false negatives can result in further transmission of infection from individuals not being referred for further assessments. Therefore, testing strategies have been devised to enhance the PPV and diagnostic accuracy of the reported results. The two-assay testing strategy could reduce false positives and improve the PPV to almost 100%, even in extremely low-prevalence settings [24]. In this study, seven false qualitative HBsAg results were obtained, where three were false negatives and four were false positives. During the follow-up period, the seven individuals with equivocal results showed consistent serologic HBV-associated test results with the initial test results. In addition, the inconsistent qualitative HBsAg results were interpreted as negative or positive. Of the three false negatives, two samples (IDs 271 and 299) showed HBsAg loss/seroconversion during antiviral therapy because they had near-quantitative LoQ levels of 0.05 and 0.08, respectively. In adult patients with CHB, the serum HBsAg levels have better predictive values for favorable outcomes, although higher serum HBV DNA levels are associated with the development of cirrhosis and hepatocellular carcinoma (HCC). In spontaneous HBeAg seroconverters with HBV genotype B or C infections, lower serum HBsAg levels in the early HBeAg-negative phase are associated with higher rates of HBsAg losses, and HBsAg cutoff levels <100 IU/mL are appropriate for predicting HBsAg losses over time [25]. The majority of Korean patients are infected with genotype C HBV [26]. Therefore, even after HBsAg seroclearance, patients with these risk factors require HCC surveillance [22]. As per the serologic test results, the third false-negative sample (ID 285) seemed to be an active CHB infection, even though HBV DNA was not quantitated. Of the four individuals falsely diagnosed as positive, three individuals (sample IDs 28, 309, and 362) did not experience HBV infections because of a lack of anti-HBs and anti-HBc IgG/IgM. Interestingly, one of these individuals (sample ID 28) was diagnosed with AHC, confirmed by the hepatitis C virus (HCV) RNA quantitation assay. However, after treatment for the HCV infection, during the follow-up period, the individual showed false-positive HBsAg results. The fourth false-positive individual (sample ID 257) had experienced an HBV infection in the past and acquired HBV immunity with anti-HBs and anti-HBc IgG.

**Figure 2:**
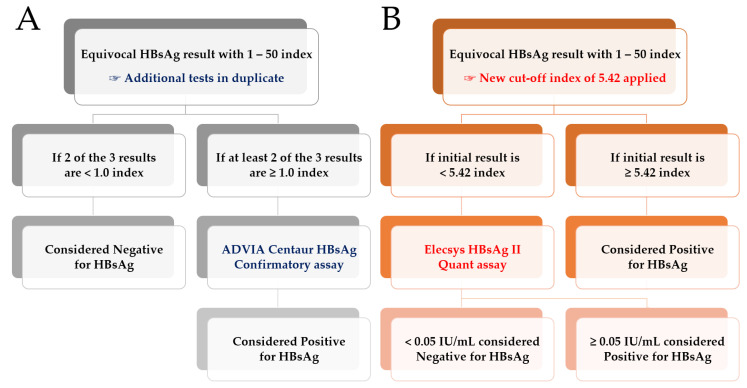
Comparison of the qualitative HBsAg testing strategy workflow for determining equivocal HBsAg results, as measured using the ADVIA Centaur HBsAg II assay. (**A**) Conventional strategy with duplicate tests; the ADVIA Centaur HBsAg Confirmatory assay is required if at least 2 out of 3 results are ≥1.0. (**B**) Proposed strategy with the new cutoff index of 5.42 without additional/duplicate tests; the Elecsys HBsAg II Quant assay is required if the initial result is <5.42.

In this study, we proposed a new two-assay testing strategy with new a COI of 5.42 without additional duplicate tests. The Elecsys HBsAg II Quant assay is required if the initial result has an index <5.42. In the proposed strategy, we observed that the cost per test of the final qualitative HBsAg result was $23.81, which is at least 33% lower than that of the conventional strategy ($31.68). With a specificity of 100%, about 78 samples (17%, 78/449) with index value ≥5.42 were interpreted as positives without the confirmatory test, and the remaining 371 samples with index value <5.42, required the Elecsys HBsAg II Quant assay. However, in the conventional strategy, additional duplicate tests had to be performed unconditionally to confirm the HBsAg results. Moreover, the ADVIA Centaur HBsAg Confirmatory assay used as a neutralization test also requires more hands-on time compared to the proposed strategy. On comparing the two HBsAg testing strategies, we comprehend that the proposed strategy involving a new COI value and the use of the quantitative HBsAg assay may be able to detect more true positives, thereby obviating the need for a confirmatory test, which is more pertinent in a resource-constrained setting (Figure 2). In a similar reference study to reduce the cost and time as well as simplify the test process, the appropriate cutoff value of the COI was found to be 6.0 for the Elecsys HBsAg II assay and a confirmatory test was required [27]. In the new laboratory workflow we have suggested in this study, we expect an increase in PPV in weakly reactive specimens as well as lowered time and cost with the elimination of the need for unnecessary confirmatory testing.

There are some inherent limitations to this study. (1) We did not perform additional serologic anti-HBs Ab, HBeAg, anti-HBe Ab, and anti-HBc IgG/IgM Ab tests as well as HBV DNA quantitation to confirm the concordant HBsAg results and to resolve the discordant HBsAg results at the same time in some leftover samples, even though all studied samples were reviewed using patients’ clinical history and serologic test results using EMR and LIS during the followed-up period. Although rare, the possibility that the concordant HBsAg results were false positives or false negatives cannot be ruled out. (2) All samples were tested initially with the ADVIA Centaur HBsAg II assay, and subsequent tests were performed only on the initially reactive samples, though additional different samples should have ideally been resubmitted to a medical laboratory for retests. (3) Only samples with index value ranging from 1 to 50 at the time of initial testing were selected. Samples that tested negative near COI values <1.0 in the initial testing with the ADVIA Centaur HBsAg II assay were not subjected to the conventional tests but were subjected to the proposed test strategy Therefore, the false-negative rates could have been underestimated. Although it is normal to have a lower limit for taking samples for confirmatory tests, the proposed strategy could have a better impact if a further study can include at least a few of these samples by lowering the cutoff COI values, depending on the availability of samples. (4) The samples submitted to the medical laboratory by outpatients and inpatients for HBsAg testing were selected relatively intermediately from the high-prevalence population; therefore, the results generated from this potential patient population cannot be applied to the general population. This study suggests the use of a combined test strategy comprising two quantitative and qualitative HBsAg assays with a new qualitative COI value. Our proposal may be further evaluated and confirmed through larger studies. (5) The HBV DNA quantitation test, as the gold standard method, was not used to confirm the possibility of HBV presence in the studied leftover samples, even though our secondary goal was to assess the utility of the Elecsys assay compared to that of the qualitative HBsAg assay as a screening test in resolving equivocal qualitative HBsAg results in the medical laboratory without duplicate HBsAg qualitative tests or additional serologic tests. In the real world, ambiguous qualitative HBsAg test results must be confirmed by HBV DNA quantitation, regardless of the serum HBsAg level.

## 5. Conclusions

In this study, the Elecsys HBsAg II Quant assay demonstrated a highly sensitive LoQ of 0.05 IU/mL and linearity results based on the WHO IS material. We confirm that the Elecsys HBsAg II Quant assay is an accurate and sensitive in vitro diagnostic test and therapeutic strategy for HBV infection. Further, we recommend it as an alternative confirmatory HBsAg assay to the conventional strategy for resolving equivocal qualitative HBsAg results without duplicate HBsAg qualitative tests.

## Figures and Tables

**Table 1:** Comparison of performance characteristics of two HBsAg assays provided by the manufacturer.

Performance Characteristics	Elecsys HBsAg II Quant Assay	ADVIA Centaur HBsAg II Assay
Manufacturer	Roche Diagnostics	Siemens Healthcare Diagnostics
Principle of operation	ECLIA	CLIA
Measurement/Unit	Quantitative/IU/mL	Qualitative/index
Capture antibody	Biotinylated monoclonal (mouse)	Biotinylated monoclonal (mouse)
Conjugate antibody	Ruthenium complex polyclonal (sheep)	Acridinium-ester-labeled monoclonal (mouse)
Duration of the assay (min)	18	23
Sample volume (μL)	50	100
Limit of quantitation (IU/mL)	≥0.05	0.040 at the 1.0 index cutoff
Precision	CV <3.2% in Cobas e601/e602	CV <3.6% in ADVIA Centaur XP
Analytical measuring range (theoretical)	0.05–130 IU/mL (pre-dilution applied) *	0.1–1000 index
Traceability of the value assigned to the calibrator	WHO International Reference Standard, 00/588	WHO International Reference Standard, 00/588

ECLIA, electrochemiluminescence immunoassay; CLIA, chemiluminescence immunoassay. * 5–13,000 IU/mL for 100-fold diluted samples in Elecsys 2010 and Cobas e411 analyzers. 20–52,000 IU/mL for 400-fold diluted samples in Cobas e601/e602.

**Table 2:** Serologic test results of seven discordant HBsAg samples measured by Elecsys HBsAg II Quant and ADVIA Centaur HBsAg II assays.

Sample ID	HBsAg Qual 1st	HBsAg Qual 2nd	HBsAg Qual 3rd	Qual Result	Quant Result	Anti-HBs	HBeAg	Anti-HBe	Anti-HBc IgG	Anti-HBc IgM	HBV DNA	Diagnosis
271	1.19	0.11	0.24	Neg	0.05	Pos	Neg	Neg	Pos	Neg	ND	CHB
285	1.05	<0.1	<0.1	Neg	116.04	Pos	Pos	Neg	Pos	Neg	na	CHB
299	1.02	0.80	0.87	Neg	0.80	Pos	Neg	Pos	Pos	Neg	ND	CHB
28	1.08	0.78	1.02	Pos	<0.05	Neg	Neg	Neg	Neg	Neg	ND	AHC
257	1.50	1.02	1.01	Pos	<0.05	Pos	Neg	Pos	Pos	Neg	ND	NVH
309	3.34	3.59	3.24	Pos	<0.05	Neg	Neg	Neg	Neg	Neg	ND	NVH
362	3.52	3.63	3.38	Pos	<0.05	Neg	Neg	Neg	Neg	Neg	ND	NVH

Qual, HbsAg qualitative result measured by the ADVIA Centaur HBsAg II assay; Quant Result, HbsAg quantitative result measured by the Elecsys HBsAg II Quant assay; ND, not detected; na, not available; CHB, chronic hepatitis B; AHC, acute hepatitis C; NVH, non-viral hepatitis.

**Table 3:** Diagnostic performance statistics of Elecsys HBsAg II Quant and ADVIA Centaur HBsAg II assays in 449 equivocal samples.

Statistics	Elecsys HBsAg II Quant Assay	ADVIA Centaur HBsAg II Assay
Kappa agreement	Almost perfect (Kappa, 0.9669; 95% CI, 0.9426 to 0.9912)	
Concordance rate (%)	100	98.44
True positive (*n*)	170	167
True negative (*n*)	279	275
False positive (*n*)	0	4
False negative (*n*)	0	3
Sensitivity (%)	100 (95% CI, 97.87 to 100)	98.24 (95% CI, 94.93 to 99.63)
Specificity (%)	100 (95% CI, 98.68 to 100)	98.57 (95% CI, 96.37 to 99.61)
Positive likelihood ratio	Not available	68.52 (95% CI, 25.89 to 181.32)
Negative likelihood ratio	0 (95% CI, not available)	0.02 (95% CI, 0.01 to 0.05)
Positive predictive value * (%)	100 (95% CI, not available)	79.18 (95% CI, 58.98 to 90.96)
Negative predictive value * (%)	100 (95% CI, not available)	99.90 (95% CI, 99.70 to 99.97)
Accuracy * (%)	100 (95% CI, 99.18 to 100)	98.55 (95% CI, 96.96 to 99.44)

* HBV surface antigen (HBsAg) seroprevalence of 5.26% in the Western Pacific region, including the Korea, Japan, China, Philippines, and Vietnam.

## Data Availability

Not applicable.
